# CK-3, A Novel Methsulfonyl Pyridine Derivative, Suppresses Hepatocellular Carcinoma Proliferation and Invasion by Blocking the PI3K/AKT/mTOR and MAPK/ERK Pathways

**DOI:** 10.3389/fonc.2021.717626

**Published:** 2021-07-28

**Authors:** Qiong Wu, Tian-yi Liu, Bai-chun Hu, Xiang Li, Yu-ting Wu, Xiao-tong Sun, Xiao-wen Jiang, Shu Wang, Xiao-chun Qin, Huai-wei Ding, Qing-chun Zhao

**Affiliations:** ^1^Department of Pharmacy, General Hospital of Northern Theater Command, Shenyang, China; ^2^Department of Life Science and Biochemistry, Shenyang Pharmaceutical University, Shenyang, China; ^3^Department of Traditional Chinese Medicine, Shenyang Pharmaceutical University, Shenyang, China; ^4^Key Laboratory of Structure-Based Drug Design and Discovery of Ministry of Education, Shenyang Pharmaceutical University, Shenyang, China

**Keywords:** hepatocellular carcinoma, proliferation, PI3K/AKT/mTOR, MAPK/ERK, apoptosis

## Abstract

Hepatocellular carcinoma (HCC) is an aggressive tumor with a poor prognosis that highly expresses phosphatidylinositol 3-kinase (PI3K) and mitogen-activated protein kinase (ERK). The PI3K/AKT/mTOR and MAPK/ERK signaling pathways play a crucial role in HCC tumor formation, cell cycle, apoptosis and survival. However, no effective targeted therapies against these pathways is available, mainly due to the extensive and complex negative feedback loops between them. Here we used CK-3, a dual blocker of the PI3K/AKT/mTOR and MAPK/ERK pathways, against HCC cell lines to verify its anti-tumor activity *in vitro*. CK-3 exhibited cytotoxic activity against HCC, as demonstrated with MTT and colony formation assays. The anti-metastatic potential of CK-3 was demonstrated with wound healing and cell invasion assays. The ability of CK-3 to block both the PI3K/AKT/mTOR and MAPK/ERK pathways was also confirmed. CK-3 induced the apoptosis of Hep3B cells, while Bel7402 cells died *via* mitotic catastrophe (MC). Oral administration of CK-3 also inhibited the subcutaneous growth of BEL7402 cells in nude mice. Simultaneous PI3K/AKT/mTOR and MAPK/ERK pathway inhibition with CK-3 may be superior to single pathway monotherapies by inhibiting their feedback-regulation, and represents a potential treatment for HCC.

## Introduction

Liver cancer is one of the leading causes of cancer-related deaths worldwide (1–3). Hepatocellular carcinoma (HCC) accounts for the vast majority of liver cancers, representing nearly 75% of all primary liver tumors (4–6). The geographic regions with the highest incidence of HCC are Asia and Africa, and nearly half of all cases are estimated to occur in China (7–10). The most effective treatment for liver cancer is presently surgical resection. However, this treatment can only be performed when the liver has sufficient intrinsic function, therefore it is not an option for patients with cirrhosis (11–14). Understanding the molecular mechanisms of HCC oncogenesis is therefore critical to treating life-threatening liver cancers in patients who are not surgical candidates (15, 16).

The PI3K/AKT/mTOR signaling pathway plays a crucial role in tumor formation, cell cycle progression, apoptosis, and survival (17–20). The development of targeted therapies against these pathways has not been entirely successful, mainly because of the extensive and complex internal and external pathway negative feedback loops between them (21). PI3K (phosphatidylinositol 3-kinase) is activated by a variety of mitotic signals and catalyzes the formation of secondary lipid messenger phosphphao-tidylinositol-3,4,5-triphosphate (22–24). The mTOR protein kinase includes two distinct protein complexes that collectively regulate the PI3K/AKT/mTOR signaling pathway (25). AKT directly phosphorylates mTOR, which in turn phosphorylates P70S6K, a downstream substrate of mTOR that is critical to protein synthesis (26). Previous studies hypothesized that tumor resistance to MAPK/ERK (mitogen-activated protein kinase/extracellular signal-regulated kinase) inhibition may be the result of a negative feedback loop formed by the activation of AKT in response to ERK inhibition (27–29). There is also crosstalk between the PI3K and MAPK pathways. A negative feedback loop occurs after ERK suppression, activating the PI3K pathway (30–32). This loop is likely related to the mechanism by which the oncogenic Ras protein activates the MAPK/ERK signaling cascade towards mTOR activation (33). Hepatocellular carcinoma (HCC) is an aggressive tumor with a poor prognosis that highly expresses phosphatidylinositol 3-kinase (PI3K) and mitogen-activated protein kinase (ERK) (34, 35). Given these obstacles, the combined inhibition of both the PI3K/AKT/mTOR and MAPK/ERK pathways may be a potential strategy for treating liver cancer.

**Figure 1:**
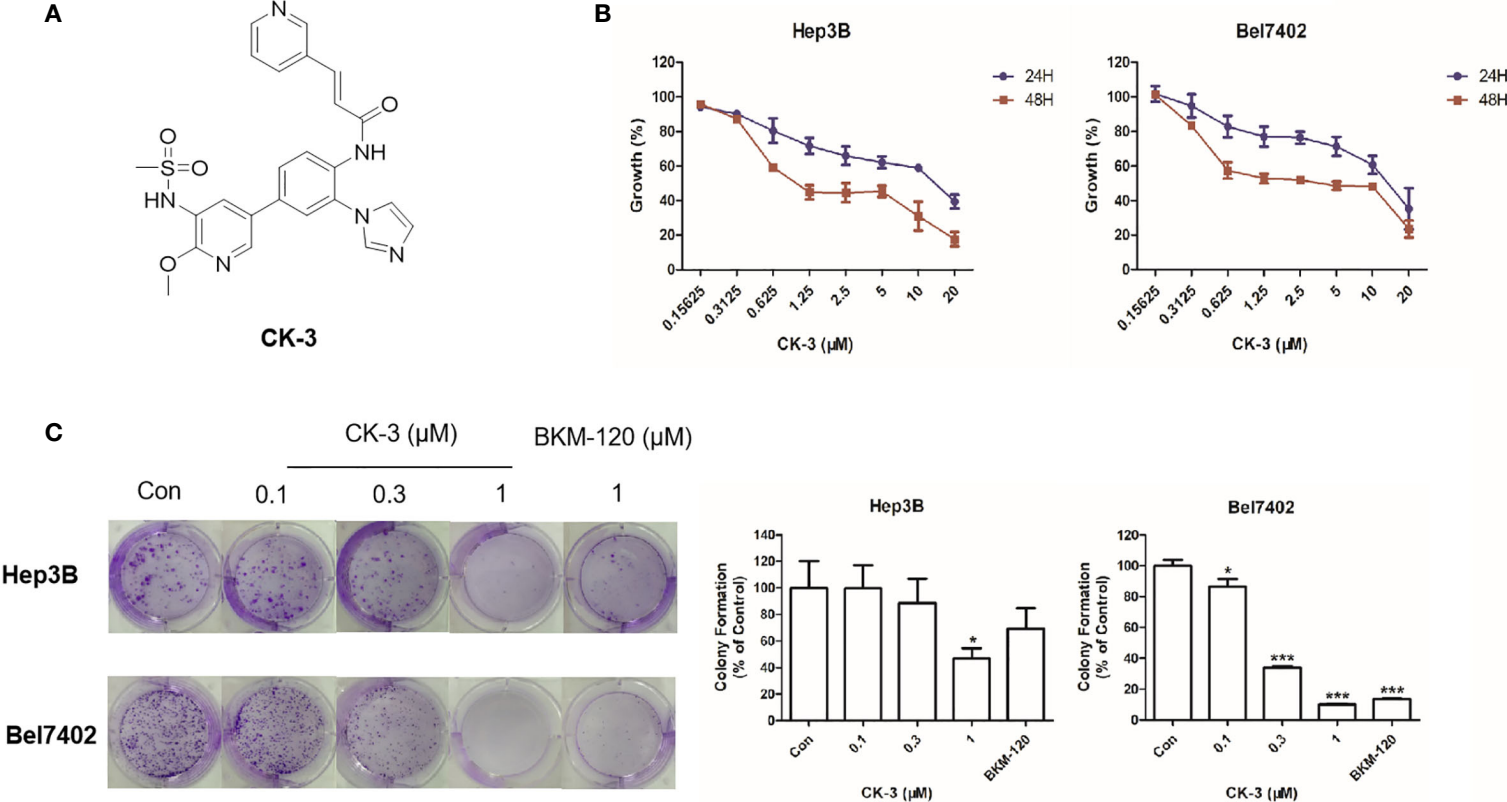
The effect of CK-3 on Hep3B and Bel7402 cell proliferation. **(A)** Structure of CK-3. **(B)** MTT assay of cells treated with CK-3 for 24, 48, and 72 h. **(C)** Effect of CK-3 on cell proliferation as evaluated with the colony formation assay. Data are presented as the mean ± SD of three independent experiments (n = 3). *p < 0.05, ***p < 0.001 compared with the control group.

In this study we investigated the anti-cancer properties of CK-3 (**Figure 1A**), a dual inhibitor of the PI3K/AKT/mTOR and MAPK/ERK pathways, on multiple HCC cell lines. CK-3 can function as a potential treatment of HCC.

## Methods and Materials

### Chemical Compounds and Reagents

CK-3 was provided by associate professor Ding Huaiwei (Shenyang Pharmaceutical University) (**Supplemental Figure 1**). Purity (99.1%) was verified with high performance liquid chromatography. The molecular structure of CK-3 was analyzed with proton nuclear magnetic resonance and mass spectrometry (**Supplemental Figures 2–4**). The small molecular inhibitor of PI3K/AKT pathway (LY294002) or MEK (GSK2118436) was conserved in our lab. For the cellular experiments, DMSO (dimethyl sulfoxide) was used to dissolve the pure CK-3 powder (also the other agents), and then use DMEM (DuIbecco’s modified eagIe’s medium) without FBS (Fetal Bovine Serum) to dilute the CK-3 (also the other agents) DMSO-solution to prepare solutions containing a series of concentrations CK-3. For the animal experiments, PEG400 and Tween 80 was used to dissolve the pure CK-3 powder, and then use physiological saline to dilute the CK-3 PEG400-Tween 80-solution to prepare solutions containing a series of concentrations CK-3. These two solutions/samples were prepared according to the methods descripted by Xie at al. (36) and Wang et al. (36, 37).

### Cell Culture

The Hepatocellular carcinoma cell lines, MHCC97-H, Hep3B, HepG2, Bel7402 and SMMC7721, were obtained from the American Type Culture Collection (ATCC). Cells were cultured in Dulbecco’s Modified Eagle Medium (Logan, UT, USA) with 10% fetal bovine serum (FBS) (Invitrogen, Carlsbad, CA, USA) at 37°C and 5% CO_2_ condition.

### Cell Survival Examination

Cell viability was assessed with a routine MTT assay (7, 38). Cells were seeded in 96-well plates in complete medium. After being incubated overnight they were exposed to diverse concentrations of CK-3 for 24 h, 48 h and 72 h. The cells were then analyzed using the MTT (0.5 mg/ml) assay and measured with a microplate-reader (Elx 800 Bio-Tek, USA). Cell viability was expressed as a percentage, with comparisons between the IC_50_ values of anti-tumor compound on HCC cells *vs*. controls.

### Colony Formation Assay

Hep3B and Bel7402 cells were seeded into 6-well plates with a density of 1×10^3^ cells/well and treated with CK-3 (0.1 [low concentration], 0.3 [middle concentration] and 1 [high concentration] μM) for 48 h. After being shifted into fresh medium without drug treatment for 1 week, the colonies were washed twice with phosphate-buffered saline (PBS) and fixed with 4% paraformaldehyde (PFA) for 10 min. The colonies were then stained with crystal violet (Beyotime, Nanjing, China) and quantified after being dissolved with glacial acetic acid (38). The plates were then analyzed with a microplate reader.

### Wound-Healing Approach

Hep3B (3×10^5^/ml) and Bel7402 cells (5×10^5^/ml) were seeded into 6-well plates. Confluent cells were scraped across the diameter of the well with a 200-μL pipette tip (39). The cells were washed twice with PBS. The migration ability of the cells was tested after CK-3 (0.3, 1 and 3 μM) treatment for 48 h. Wound area at 0 h and 48 h was measured with NIH ImageJ (Bethesda, MD, USA).

### Cell Invasion and Migration Assay

The invasiveness (the *in vitro* invasion examination) of Hep3B and Bel7402 cells after treatment with CK-3 (0.3, 1 and 3 μM) was evaluated with a 24-well Transwell Matrigel (Corning Life Sciences, Bedford, MA, USA). Cells were seeded into the upper chambers of the Matrigel after being suspended in serum-free medium. Medium containing 10% FBS was added to the lower chambers. After 48 h, cells that penetrated into the lower chambers were fixed with 4% paraformaldehyde (PFA) for 10 min, stained with 0.1% crystal violet solution for 30 min, then washed twice with PBS. For the cell migration assay, the cells were directed seeded in to the upper chambers. The wells were quantified and the relative invasion/migration cell numbers were calculated according to the methods by Yu et al. (40).

### *In Vitro* Phosphatidylinositol 3-Kinase (PI3K) Inhibition Assay

This experiment was performed by Shanghai ChemPartner Co., Ltd. The PI3K assay was performed with the PI3K-GloTM Class Profiling Kit (Promega, #V1690), which measured the amount of ADP produced during the kinase reaction. IC_50_ values were compared with Prism.

### Molecular Docking

The X-ray crystal structures of PI3K (PDB ID code: 3DBS) and ERK (PDB ID code: 5K4I) were obtained from the Protein Data Bank (PDB) (http://www.wwpdb.org) and prepared with the protein preparation wizard in Maestro (version 10.6, Schrödinger, Cambridge, MA, USA). The active site was defined according to the binding position of the co-crystal ligand. Glide was used for protein-ligand docking in standard precision (SP) after ligand preparation.

### Western Blotting

Total protein samples were extracted from Hep3B and Bel7402 cells, separated with sodium dodecyl sulfate polyacrylamide gel electrophoresis (SDS–PAGE), and electrophoretically transferred to polyvinylidene fluoride (PVDF) membranes (Millipore, Billerica, MA, USA). The membranes were then blocked with non-fat milk (5% in TBST) at 4°C overnight, and the primary antibody was incubated for 2 h at room temperature. After washing three times with TBST (10 min for each time), the membranes were incubated with horseradish peroxidase (HRP) conjugated secondary antibodies for 2 h at room temperature. Blots were visualized with enhanced chemiluminescence reagents and analyzed using ImageJ.

The following primary antibodies were purchased from Cell Signaling Technology (Danvers, MA, USA): E-cadherin, PTEN, p-AKT (Ser473), AKT, p-mTOR, mTOR, p-p70S6K1 (Thr389), p70S6K1, p-ERK1/2, ERK1/2, Bcl-2, and Bax. The antibody against GAPDH was purchased from Proteintech (Rosemont, IL, USA). Anti-rabbit IgG and anti-mouse IgG secondary antibodies conjugated with horseradish peroxidase (HRP) were purchased from Abcam (Cambridge, UK).

### Cell Morphology Changes

Hep3B and Bel7402 cells were treated with CK-3 for 48 h, then stained with Hoechst 33342 (Beyotime, Shanghai, China). After being washed twice with PBS the cells were observed and photographed with a fluorescence microscope (Olympus, Tokyo, Japan).

### Apoptosis Analysis

After treatment with CK-3 for 48 h, Hep3B and Bel7402 cells were collected and washed twice with PBS. The cells were then incubated with Annexin V-FITC and PI from the Annexin V-FITC Apoptosis Kit (BD, Pharmingen, USA) in the dark for 20 min as per the manufacturer’s instructions. The cells were then re-suspended in binding buffer and measured with flow cytometry analysis (FACS) (BD Biosciences, Franklin Lakes, NJ, USA). Data was analyzed with Flow Jo.7.6.1 (Tree Star, Ashland, OR, USA) (41, 42).

### Cell Cycle Analysis

After incubation with CK-3 for 48 h, Hep3B and Bel7402 cells were collected and fixed overnight in cold 70% (v/v) ethanol at 4°C. After being washed with PBS the cells were stained with PI using the Cell Cycle Analysis Kit (BD) according to the manufacturer’s instructions. The samples were then analyzed with fluorescence-activated cell sorting (43).

### The *In Vivo* Antitumor Activation of CK-3 *via* a Nude Mice Model

The usage of nude mice were permitted by the Animal Ethics Committee, General Hospital of Northern Theater Command. BEL7402 cells were cultured and injected into the nude mice mice’s subcutaneous tumor position. Mice were received 10mg/kg (high dose), 5mg/kg (middle dose) or 1mg/kg (low dose) of CK-3 *via* oral administration once per two days. After 10-15 times’ treatment, the tumors were collected and the tumor volumes/tumor weights were examined (25, 44, 45). The inhibitory rates of CK-3 on BEL-7402 cells’ subcutaneous growth was calculated according to the tumor volumes or tumor weights. The expression level of cell proliferation related factor Ki67; the EMT (epithelial-mesenchymal transition) related factors N-Cadherin, E-Cadherin, Vimentin; the pro-survival/anti-apoptosis related factors Survivin, cIAP-1, cIAP-2, BCL-2 in the tumor tissues was examined by qPCR (quantitative polymerase chain reaction) following the methods descripted by Ma et al. (46) and Yang et al. (10), and the primers were also used the sequences provided by Ma et al. (46). The heat-map was performed according the methods descripted by Zhou et al. and Wang et al. (29, 47).

### Statistical Analysis

All data were expressed as mean ± SD, and all experiments were performed in triplicate. Differences among experimental groups were compared using analysis of variance (ANOVA) followed by the Student’s *t*-test (P < 0.05). SPSS22.0 (IBM, Armonk, NY, USA) was used for statistical analysis and GraphPad software (GraphPad Software, La Jolla, CA, USA) was used to present the analyzed data. p-values < 0.05 were considered to be statistically significant.

## Results

### CK-3 Has a Cytotoxic Effect Against Various HCC Cell Lines

The MTT assay showed that CK-3 had a cytotoxic effect against HCC cell lines, especially Hep3B and Bel7402 cells (**Table 1**). Testing different concentrations of CK-3 for 24 h, 48 h and 72h (**Figure 1B**) demonstrated that the effect of CK-3 on cell proliferation was time- and dose-dependent. Moreover, the colony formation assay indicated that CK-3 can reduce the proliferation of Hep3B and Bel7402 cells (**Figure 1C**).

**Table 1:** The antiproliferative activity of CK-3 on HCC cell lines (mean ± SD, n=3).

Cell line	IC_50_ (μM)
HepG2	4.670 ± 4.316
SMMC7721	5.210 ± 3.886
MHCC97-H	3.053 ± 0.659
Bel7402	1.556 ± 0.606
Hep3B	1.358 ± 0.309

### CK-3 Inhibits the Migration and Invasion of HCC Cell Lines

**Figure 2:**
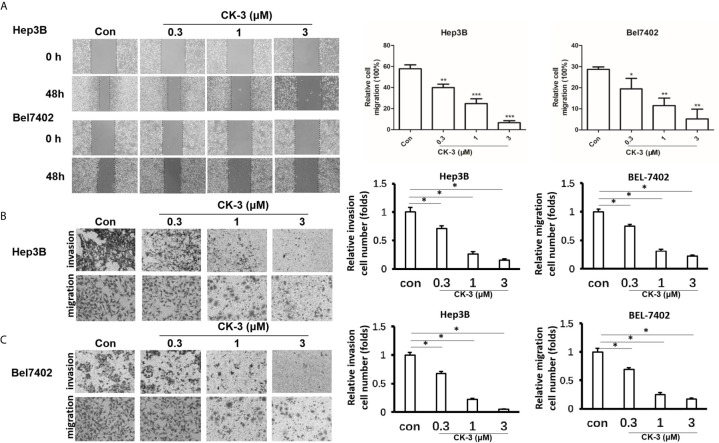
Effects of CK-3 on Hep3B and Bel7402 cell migration and invasion. **(A)** CK-3 inhibited the migration of cells as demonstrated with a wound-healing assay. Dashed lines represent the edge of a wound. Micrographs were taken at 100x magnification. **(B, C)** Inhibition effects of CK-3 on invasion/migration as demonstrated with a Transwell experiment. Stained cells were quantified by counting three random fields per Transwell. Micrographs were taken at 200x magnification. *p < 0.05, **p < 0.01, ***p < 0.001, compared with controls.

We evaluated the migration capabilities of HCC cell lines after CK-3 treatment using a wound healing assay. The number of cells that migrated into a wounded region after treatment with CK-3 for 48 h were counted and compared with controls (**Figure 2A**). A Transwell invasion assay or transwell migration assay was then used to confirm the *in vitro* invasiveness/migration of these cells. As shown in **Figure 2B**, CK-3 notably decreased the number of cells that invaded or migrated to the bottom of the membrane. The level of E-cadherin was increased in Hep3B and Bel7402 cells after treatment with CK-3 for 48 h (**Supplemental Figure 5**). The dose dependency was also noted by CK-3’s effect (**Figure 2** and **Supplemental Figure 5**).

### CK-3 Suppresses HCC Cell Lines by Down-Regulating Both the PI3K/AKT/mTOR and MAPK/ERK Signaling Pathways

**Figure 3:**
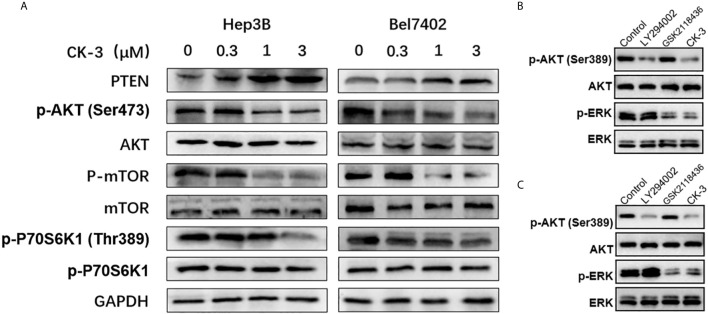
CK-3 suppressed the PI3K/AKT/mTOR and MAPK/ERK pathways in Hep3B and Bel7402 cells. **(A)** Inhibitory effects of CK-3 on the level of PTEN, p-AKT, AKT, p-mTOR, mTOR, p-p70S6K and p70S6K proteins as shown with western blotting. **(B, C)** The inhibitory effects of CK-3, LY294002 or GSK2118436 on the level of p-ERK and EKK proteins as shown with western blotting.

To investigate whether CK-3 inhibited Hep3B and Bel7402 cell proliferation by regulating PI3K, we examined the ability of CK-3 to inhibit PI3K activity *in vitro* (**Table 2**). CK-3 selectively inhibited the activity of the PI3K catalytic subunits PI3Kα and PI3Kδ. As indicated in **Figure 3A**, the expression of p-AKT (Ser473 point), p-mTOR and p-p70S6K1 (Thr389) was decreased after CK-3’s treatment. In addition, CK-3 treatment resulted in a significant increase in the expression of PTEN, which is an AKT pathway inhibitor (**Figure 3A**). Moreover, CK-3 could also inhibited the phosphorylation of ERK in HCC cells (**Supplemental Figure 6**). The specificity of CK-3 on HCC cells were confirmed by using the LY294002 or GSK2118436 in HCC cells (**Figures 3B, C**).

**Table 2:** PI3 Kinase-activity profile of CK-3.

Kinase	IC_50_ (μM)
PI3Kα	6.709
PI3Kβ	>10
PI3Kδ	4.671
PI3Kγ	>10
ERK	5.667

**Figure 4:**
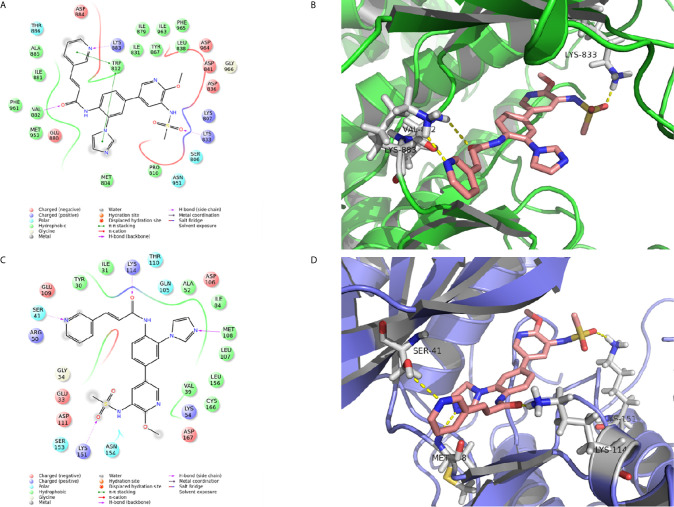
The molecular docking of CK-3 with PI3K or ERK. **(A, C)** A two-dimensional interaction diagram of CK-3 on the catalytic cavity of PI3K (PDB code: 3DBS) and ERK (PDB code: 5K4I). **(B, D)** A three-dimensional interaction diagram of CK-3 on the catalytic cavity of PI3K and ERK. The crystal structures of proteins are depicted as green and purple cartoons, and key amino acids are depicted as gray sticks. CK-3 is shown as pink sticks. Hydrogen bonds are represented by yellow dotted lines.

The interaction between proteins and CK-3 was explored with molecular docking technology. As shown in **Figures 4A, B**, the pyridine ring of CK-3 penetrated into the active site of PI3Kγ and formed a major hydrogen bond with Lys883. Moreover, Lys833 and Val882 also formed hydrogen bonds with the oxygen atom on the sulfonyl group and the oxygen atom on the sidechain. Furthermore, the optimal conformation of CK-3 was well matched to the ERK2 protein active cavity (**Figures 4C, D**). The oxygen atom of the amide on the sidechain and the nitrogen atom on the pyridine ring formed hydrogen bonds with Lys114 and Ser41 as hydrogen acceptors. Met108 and Lys151 also interacted with the nitrogen atom on the imidazole ring and the oxygen atom on the sulfonyl group with hydrogen bonds.

### CK-3 Effects on Apoptosis and Cell Cycle Arrest in HCC Cell Lines

**Figure 5:**
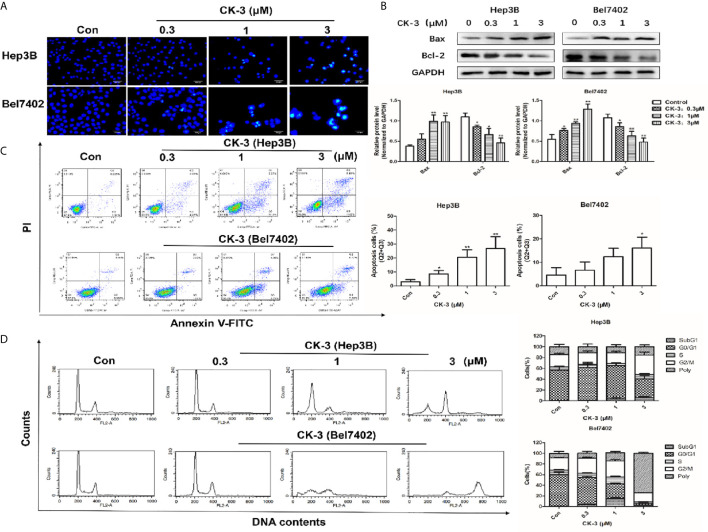
Effects of CK-3 on Hep3B and Bel7402 cell apoptosis and reproduction. **(A)** Morphology of the cell nuclei changes following incubation with CK-3 for 48 h, as visualized with Hoechst 33342. Scale bar =200 μm. **(B)** The level of Bax and Bcl-2 in cells treated with CK-3 at various concentrations for 48 h was evaluated with western blotting. All blotted proteins were normalized to GAPDH. **(C)** Annexin V-FITC/PI double-staining of cells after 48 h of CK-3 treatment. Bar graphs show the quantification of the apoptotic cells. **(D)** DNA analysis of cells after CK-3 treatment was performed using flow cytometry. The percentage of cells in each phase of the cell cycle is shown in the histogram. Data are presented as mean ± SD of three independent experiments (n = 3). *p < 0.05, **p < 0.01, compared with the control group.

Apoptotic cells were detected with the Hoechst 33342 and Annexin V-FITC/PI staining assays. Compared with the control group, the nuclear DNA in cells that received a high-dose of CK-3 exhibited more condensed and fragmented staining (**Figure 5A**). To verify these results the level of apoptosis-associated proteins was evaluated with western blotting. As shown in **Figure 5B**, the expression of Bax was significantly up-regulated after CK-3 treatment, while the level of Bcl-2 was markedly down-regulated. Hep3B and Bel7402 cells had a high degree of apoptosis-like death, as shown with the Annexin V-FITC/PI staining assay (**Figure 5C**).

Cell cycle distribution analysis indicated that CK-3 induced Hep3B cell cycle arrest in the G_2_/M phase. The proportion of polyploid Bel7402 cells strongly increased after CK-3 treatment for 48 h (**Figure 5D**), suggesting that CK-3 caused cell cycle arrest during mitosis, which was different from that of Hep3B cells.

### The *In Vivo* Antitumor Effect of CK-3 on BEL7402 Cells

**Figure 6:**
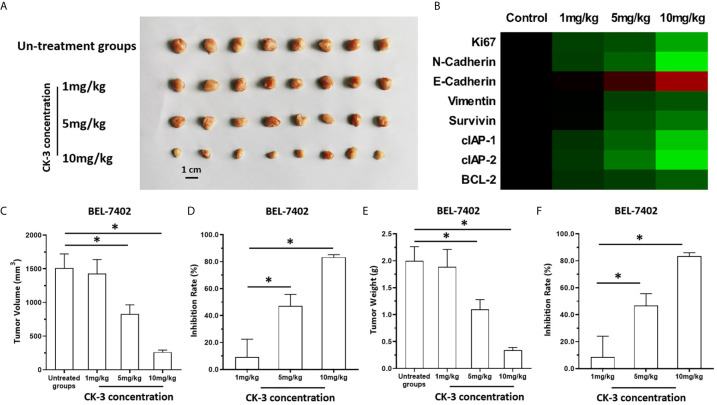
The *in vivo* antitumor activation of CK-3. BEL-7402 was cultured and injected in to the subcutaneous position of the nude mice. Mice were received the CK-3 *via* oral administration. The results were shown as the images of tumor tissues **(A)**, the heat-map indicated the inhibitory rates of CK-3 on proliferation, pro-survival, anti-apoptosis or EMT related factors **(B)**, tumor volumes **(C)**, inhibitory rates of CK-3 according to tumor volumes on Bel-7402 **(D)**, tumor weights **(E)**, inhibitory rates of CK-3 according to tumor weights on Bel-7402 **(F)**. *P < 0.05.

The above results were obtained from the cultured HCC cells. To further examine the antitumor effect of CK-3, the BEL-7402 was cultured and injected into nude mice to form subcutaneous tumor tissues. As shown in **Figure 6**, BEL-7402 cells could form subcutaneous tumor tissues in nude mice. Oral administration of 10mg/kg CK-3 (high dose of CK-3) or 10mg/kg CK-3 (middle dose of CK-3) but not 1mg/kg CK-3 (low dose of CK-3) could inhibited the subcutaneous growth of BEL-7402 cells in nude mice (**Figures 6A, C–F**). Oral administration of CK-3 also inhibited the expression of Ki67, N-cadherin, Viemntin, Survivin, cIAP-1, cIAP-2 or BCL-2 and enhanced the expression of E-cadherin in tumor tissues formed by BEL7402 cells in a dose-dependent manner (**Figure 6B**).

## Discussion

The PI3K/AKT/mTOR and MAPK/ERK pathways play a dominant role in HCC proliferation, migration, reproduction and apoptosis (48–50). Our *in vitro* experiments confirmed that CK-3 inhibited the proliferation (**Figure 1**), migration and invasion (**Figure 2**) of HCC cells by inhibiting these two pathways. Our results showed that the activity of PI3Kα and PI3Kδ was selectively reduced by CK-3 and we observed that CK-3 could simultaneously block the PI3K/AKT/mTOR and MAPK/ERK pathways. Therefore, modulation of PI3K is just one aspect of CK-3 function and it is valuable to continue to optimize the structure of CK-3. As there is a crosstalk between the PI3K and MAPK pathways ERK inhibitors may activate AKT through a negative feedback loop, thereby activating the PI3K pathway (47, 51, 52). We therefore believe that AKT feedback activation can be stabilized *via* ERK inhibition. Combination PI3K and ERK inhibition therefore seems to be a good strategy for overcoming the limitations of monotherapy, thereby best preventing AKT activation. Furthermore, a molecular modeling study showed that CK-3 can occupy the active binding sites of PI3K and ERK and form various interactions with the residues on the active site (**Figure 3**). These hydrogen bonds played a crucial role in the conformational stability of CK-3 and also increased the affinity of the compound to these proteins (53, 54).

It is well known that AKT plays an important role in the PI3K signaling pathway, which promotes the activation of a number of downstream pathways that participate in tumorigenesis (55–57). Weller (58) demonstrated that of the steps in the PI3K/AKT/mTOR cascade, mTOR and its two downstream factors 4EBP1 and P70S6K were directly activated by AKT. 4EBP1 plays a key role in cell proliferation, and P70S6K is important for mitogen integration and nutrient signaling to control cell proliferation and size (59). PTEN is an important tumor suppressor that inhibits the PI3K/AKT signaling pathway and antagonizes cell proliferation, differentiation, apoptosis, and cell cycle processes (**Figure 3**) (60, 61).

We demonstrated that CK-3 caused G2/M phase cell cycle arrest in Hep3B cells, and induced the apoptosis of these cells by regulating Bax and Bcl-2 protein expression. In contrast, CK-3 caused cell cycle arrest during the mitosis phase in Bel7402 cells, which also caused an apoptosis-like death (**Figure 4**). The major mode of programmed cell death is apoptosis, a highly regulated ordered cell destruction meant to remove damaged or redundant cells. Controlling apoptosis is an important focus of cancer treatment (62–64). Apoptosis can occur through two signaling pathways: an “external” pathway mediated by death receptors and an “internal” pathway mediated by mitochondria. Both involve mitochondria and Bcl-2 family proteins (65). Our results confirmed that CK-3 significantly down-regulated the expression of Bcl-2 and up-regulated the level of Bax, which might also be regulated by the intrinsic apoptosis pathway.

Unlike apoptosis and necrosis, MC is a novel anti-tumor process that is caused by a unique nuclear alteration. MC is considered p53-independent cell death and has the morphologic features of both apoptosis and necrosis. In our study CK-3 enlarged the volume of Bel7402 cells and increased the number of multi-nucleated cells, both of which were clearly observed by the Hoechst 33342 and Annexin V-FITC/PI double-staining assays. The formation of multinucleated cells is a typical morphological feature of MC (66). That CK-3 induced this unique form of cell death suggests that it may be useful in the setting of apoptosis resistance.

In conclusion, our results indicate that CK-3 blocks the PI3K/AKT/mTOR and MAPK/ERK signaling pathways and exerts an anti-tumor effect on Hep3B and Bel7402 cells. These findings confirm that dual targeting the PI3K/AKT/mTOR and MAPK/ERK pathways is a promising strategy for the treatment of HCC. Although CK-3 may become a candidate therapy against HCC in the future, further translational research is required.

## Data Availability Statement

The original contributions presented in the study are included in the article/**Supplementary Material**. Further inquiries can be directed to the corresponding authors.

## Ethics Statement

The presence work did not contain the patients-related materials and the usage of human related materials were permitted by the Ethics Committee of Northern Theater General Hospital. The animal experiments were permitted by the Animal Ethics Committee of Northern Theater General Hospital.

## Author Contributions

Q-cZ, H-wD, and QW conceived the main ideas and wrote the paper. X-wJ, SW, and X-cQ supervised the study. QW, T-yL and B-cH developed major methodologies, databases, reagents, and primary experiments. XL, Y-tW, and X-tS analyzed different aspects of the results. All authors contributed to the article and approved the submitted version.

## Funding

This research was supported by Liaoning Natural Fund Guidance Plan (Number: 2019-ZD-0446).

## Conflict of Interest

The authors declare that the research was conducted in the absence of any commercial or financial relationships that could be construed as a potential conflict of interest.

## Publisher’s Note

All claims expressed in this article are solely those of the authors and do not necessarily represent those of their affiliated organizations, or those of the publisher, the editors and the reviewers. Any product that may be evaluated in this article, or claim that may be made by its manufacturer, is not guaranteed or endorsed by the publisher.

## Supplementary Material

Supplementary Figure 1 The synthetic route of CK-3.

Supplementary Figure 2 The HPLC fingerprint of CK-3 in the presence work.

Supplementary Figure 3 HR-MS spectrum of compound CK-3 in the presence work.

Supplementary Figure 4 1H-NMR spectrum of compound CK-3 in the presence work.

Supplementary Figure 5 CK-3 enhanced the expression of E-Cadherin in HCC cells E-cadherin expression of HCC cell lines after treatment with CK-3 was evaluated with a western blot. All blotted proteins were normalized to GAPDH. Data are presented as the mean ± SD of three independent experiments (n = 3). *P < 0.05.

Supplementary Figure 6 CK-3 suppressed the PI3K/AKT/mTOR pathways in Hep3B and Bel7402 cells. Effects of CK-3 on p-ERK and ERK expression were evaluated. The bar graphs show the relative levels of PTEN, p-AKT, AKT, p-mTOR, mTOR, p-p70S6K, p70S6K, p-ERK, and ERK. All blotted proteins were normalized to GAPDH.

Supplementary Figure 7 CK-3 suppressed the MAPK/ERK pathways in Hep3B and Bel7402 cells. Effects of CK-3 on p-ERK and ERK expression were evaluated. The bar graphs show the relative levels of PTEN, p-AKT, AKT, p-mTOR, mTOR, p-p70S6K, p70S6K, p-ERK, and ERK. All blotted proteins were normalized to GAPD.
