# Supplementary figures and images for: CK-3, A Novel Methsulfonyl Pyridine Derivative, Suppresses Hepatocellular Carcinoma Proliferation and Invasion by Blocking the PI3K/AKT/mTOR and MAPK/ERK Pathways
Source: Front Oncol. 2021 Jul 28;11:717626. doi: 10.3389/fonc.2021.717626 (PMC8355706; doi:10.3389/fonc.2021.717626)

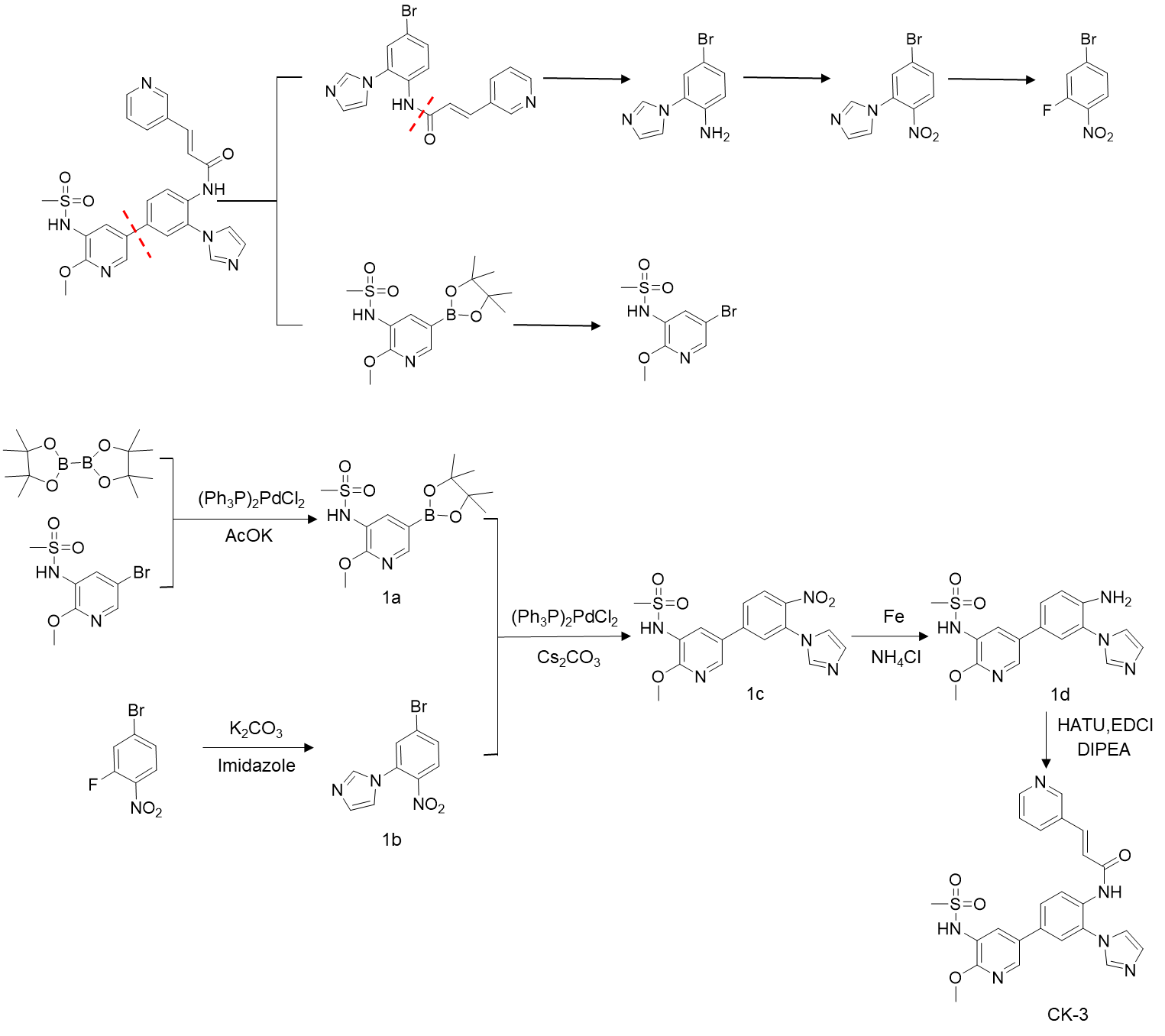


Supplemental Figure 1

Supplement: Supplementary Figure 1 — The synthetic route of CK-3. [file DataSheet_1.doc]

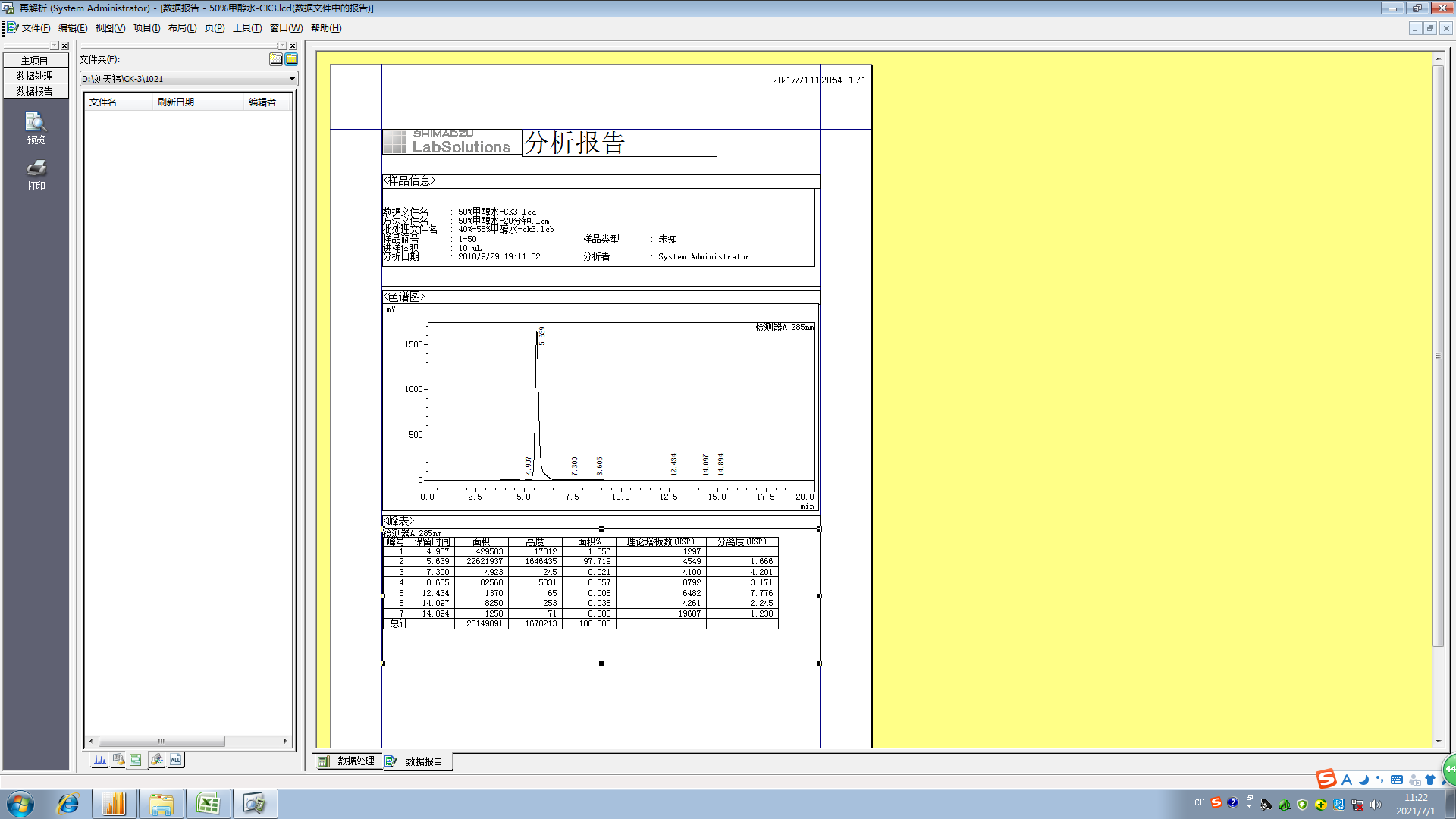


Supplemental Figure 2

Supplement: Supplementary Figure 2 — The HPLC fingerprint of CK-3 in the presence work. [file DataSheet_2.docx]

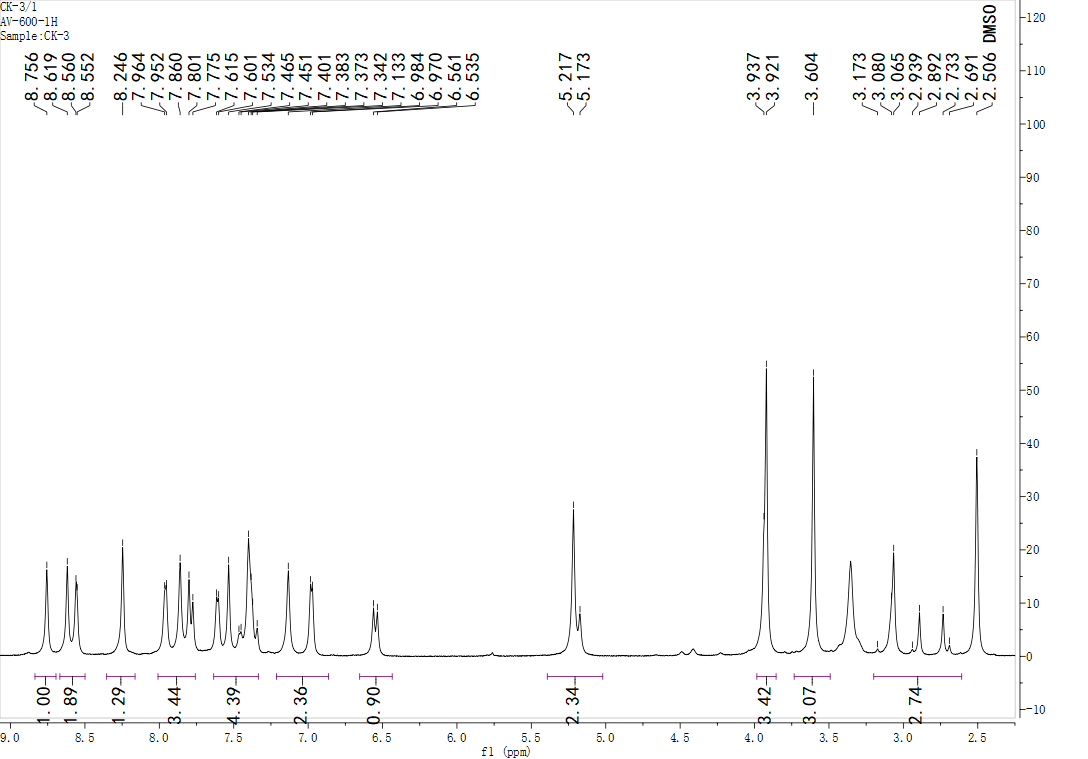


Supplemental Figure 4

Supplement: Supplementary Figure 4 — 1H-NMR spectrum of compound CK-3 in the presence work. [file DataSheet_4.docx]

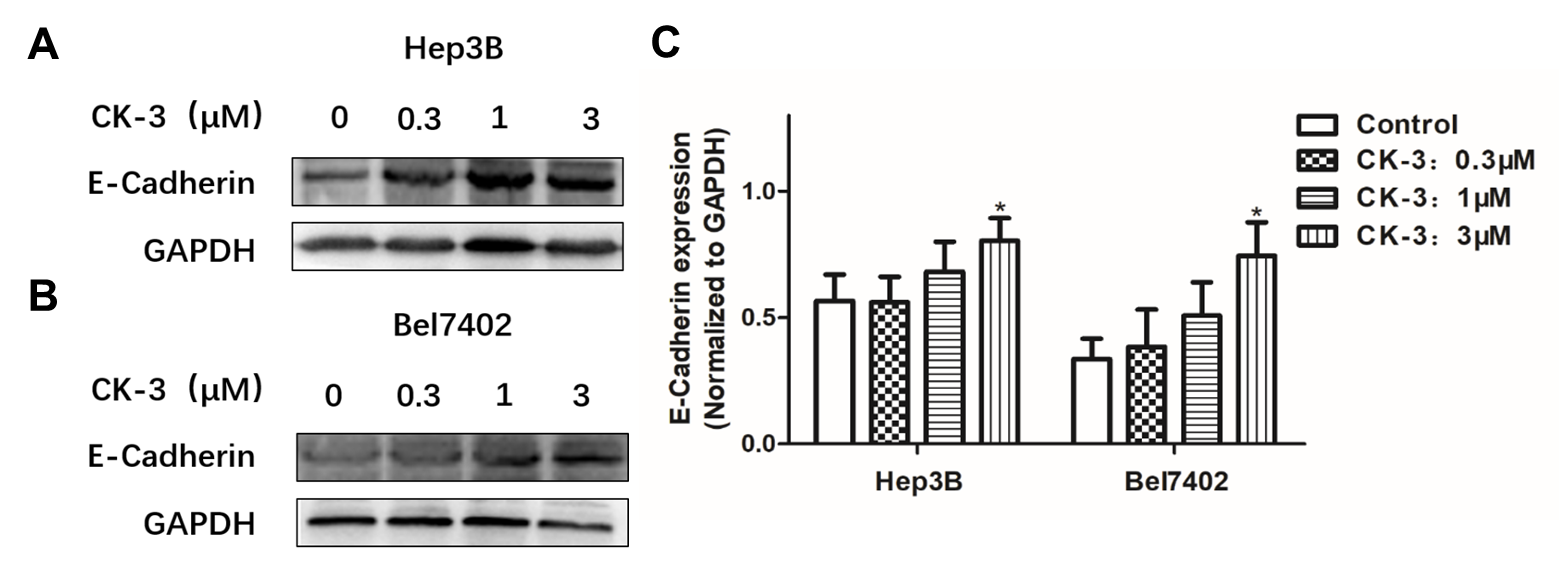


Supplemental Figure 5

Supplement: Supplementary Figure 5 — CK-3 enhanced the expression of E-Cadherin in HCC cells E-cadherin expression of HCC cell lines after treatment with CK-3 was evaluated with a western blot. All blotted proteins were normalized to GAPDH. Data are presented as the mean ± SD of three independent experiments (n = 3). *P < 0.05. [file DataSheet_5.doc]

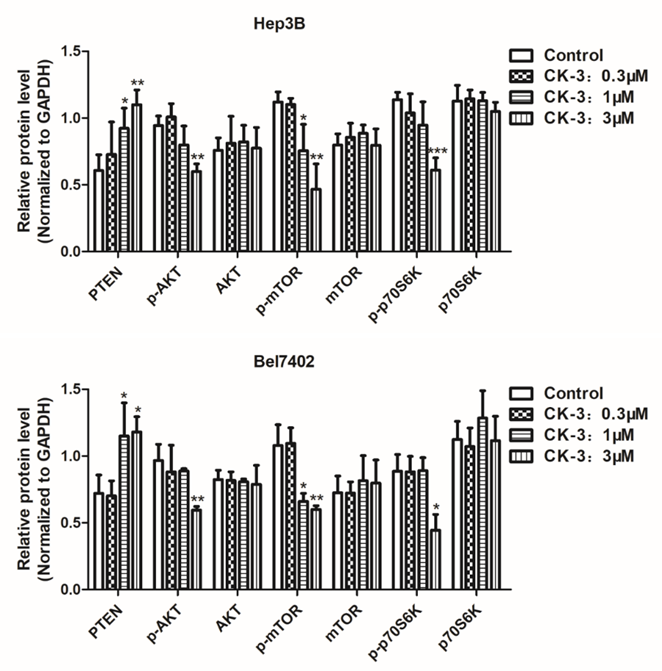


Supplemental Figure 6

Supplement: Supplementary Figure 6 — CK-3 suppressed the PI3K/AKT/mTOR pathways in Hep3B and Bel7402 cells. Effects of CK-3 on p-ERK and ERK expression were evaluated. The bar graphs show the relative levels of PTEN, p-AKT, AKT, p-mTOR, mTOR, p-p70S6K, p70S6K, p-ERK, and ERK. All blotted proteins were normalized to GAPDH. [file DataSheet_6.doc]

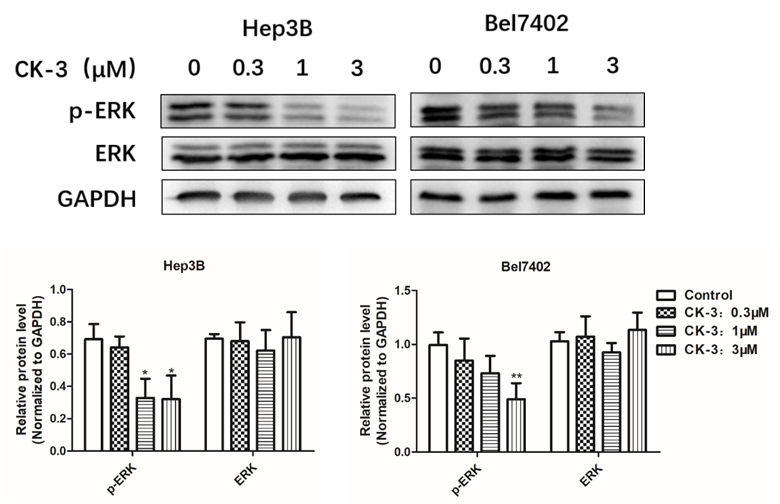


Supplemental Figure 7

Supplement: Supplementary Figure 7 — CK-3 suppressed the MAPK/ERK pathways in Hep3B and Bel7402 cells. Effects of CK-3 on p-ERK and ERK expression were evaluated. The bar graphs show the relative levels of PTEN, p-AKT, AKT, p-mTOR, mTOR, p-p70S6K, p70S6K, p-ERK, and ERK. All blotted proteins were normalized to GAPD. [file DataSheet_7.doc]
