# Supplementary material for: CK-3, A Novel Methsulfonyl Pyridine Derivative, Suppresses Hepatocellular Carcinoma Proliferation and Invasion by Blocking the PI3K/AKT/mTOR and MAPK/ERK Pathways
Source: Front Oncol. 2021 Jul 28;11:717626. doi: 10.3389/fonc.2021.717626 (PMC8355706; doi:10.3389/fonc.2021.717626)
Supplement: Supplementary Figure 3 — HR-MS spectrum of compound CK-3 in the presence work. [file DataSheet_3.docx]

Supplemental Figure 3
